# The Metabolome as a Biomarker of Health and Aging in Companion Dogs

**DOI:** 10.1093/geroni/igaf122.1392

**Published:** 2025-12-31

**Authors:** Daniel Promislow

**Affiliations:** Jean Mayer USDA Human Nutrition Research Center on Aging, Boston, Massachusetts, United States

## Abstract

The companion dog is an exceptional translational model for understanding the causes and consequences of aging. Among breeds, we observe high levels of variation not only in morphology and behavior, but also in lifespan and the risk of specific diseases, many of which are common in humans. Dogs share our environment and, like us, have a sophisticated healthcare system. Their shorter lifespan relative to humans means that lessons that might take decades to learn from human longitudinal studies can be gleaned in just a few years by observing aging in dogs. Here, we share recent findings from the Dog Aging Project, a long-term longitudinal study designed to identify the genetic and environmental factors that influence healthy aging in dogs and the mechanisms by which they do so. As part of the Dog Aging Project, we collect annual measures of the plasma metabolome for nearly 1,000 dogs from across the United States. Age-related changes in the metabolome point to underlying mechanisms that might account for breed variation in lifespan. We have also identified individual metabolites that predict mortality risk and found that the sign and magnitude of their effects are significantly correlated with patterns observed in comparable human studies. Finally, we discuss how metabolome profiles can serve as a bridge linking upstream genetic and environmental factors with downstream traits related to aging. As such, metabolome profiles provide a more complete picture of the causes and consequences of aging and related disease.

